# Comparative analyses of vertebrate posterior *HoxD *clusters reveal atypical cluster architecture in the caecilian *Typhlonectes natans*

**DOI:** 10.1186/1471-2164-11-658

**Published:** 2010-11-24

**Authors:** An Mannaert, Chris T Amemiya, Franky Bossuyt

**Affiliations:** 1 Biology Department, ECOL, Amphibian Evolution Lab, Vrije Universiteit Brussel, Brussels, Belgium; 2 Benaroya Research Institute at Virginia Mason and University of Washington, Seattle, USA

## Abstract

**Background:**

The posterior genes of the *HoxD *cluster play a crucial role in the patterning of the tetrapod limb. This region is under the control of a global, long-range enhancer that is present in all vertebrates. Variation in limb types, as is the case in amphibians, can probably not only be attributed to variation in *Hox *genes, but is likely to be the product of differences in gene regulation. With a collection of vertebrate genome sequences available today, we used a comparative genomics approach to study the posterior *HoxD *cluster of amphibians. A frog and a caecilian were included in the study to compare coding sequences as well as to determine the gain and loss of putative regulatory sequences.

**Results:**

We sequenced the posterior end of the *HoxD *cluster of a caecilian and performed comparative analyses of this region using *HoxD *clusters of other vertebrates. We determined the presence of conserved non-coding sequences and traced gains and losses of these footprints during vertebrate evolution, with particular focus on amphibians. We found that the caecilian *HoxD *cluster is almost three times larger than its mammalian counterpart. This enlargement is accompanied with the loss of one gene and the accumulation of repeats in that area. A similar phenomenon was observed in the coelacanth, where a different gene was lost and expansion of the area where the gene was lost has occurred. At least one phylogenetic footprint present in all vertebrates was lost in amphibians. This conserved region is a known regulatory element and functions as a boundary element in neural tissue to prevent expression of *Hoxd *genes.

**Conclusion:**

The posterior part of the *HoxD *cluster of *Typhlonectes natans *is among the largest known today. The loss of *Hoxd-12 *and the expansion of the intergenic region may exert an influence on the limb enhancer, by having to bypass a distance seven times that of regular *HoxD *clusters. Whether or not there is a correlation with the loss of limbs remains to be investigated. These results, together with data on other vertebrates show that the tetrapod *Hox *clusters are more variable than previously thought.

## Background

Perhaps the best studied gene clusters in animals are the *Hox *clusters, not only for their importance in the establishment of the metazoan body plan, but also for their tight genomic organization. *Hox *genes encode transcription factors that belong to the family of homeodomain proteins and play an essential role in the establishment of the anterior-posterior body axis during embryonic development. In addition, they are also involved in patterning of limbs and in organogenesis [1-4]. In vertebrates, the expression domains of *Hox *genes are collinear in space and time, and reflect their chromosomal arrangement [5].

Invertebrates possess one - often interrupted or disintegrated - *Hox *cluster, while all vertebrates have multiple clusters [6-8]. Gnathostomes typically have four *Hox *clusters that arose by subsequent duplications in the stem lineage of vertebrates, while most ray-finned fishes contain seven (e.g. zebrafish) to thirteen (e.g. salmon) clusters as a result of additional, teleost-specific genome duplications and subsequent cluster losses [9-11]. Due to these additional duplications, the gene content of the fish *Hox *clusters is variable, with different gene losses in different species examined. In contrast, the *Hox *complement of tetrapods is rather conserved, with the same genes present in mammalian and bird genomes [7]. In the genome of the frog *Silurana tropicalis*, at least one and possibly two genes have been lost [12,13].

*Hox *gene clusters in vertebrates are compact (around 100 kb in mammals and even shorter in teleost fishes), with highly conserved distances between paralogous genes, and with little or no interspersed repetitive DNA elements [14]. The only exceptions known so far are squamate reptiles, with the lizard *Anolis carolinensis *as a striking example of having accumulated a substantial number of retrotransposons in its *Hox *clusters, resulting in considerably larger cluster sizes [15,16]. In general, the tight clustering of the *Hox *genes in vertebrates may be the result of an evolutionary constraint to keep the genes in close proximity, thus maintaining the intergenic distances and prohibiting insertion of interspersed repeats [17]. This constraint may be facilitated by the presence of *cis*-regulatory elements within the clusters that are shared by neighboring genes, as well as by remote enhancers producing regulatory landscapes that would be broken when the clusters split [18]. In this context, a suite of global long-range enhancers that control the expression of six genes located 5' of the *HoxD *cluster of fishes and mammals has been discovered [19]. This Global Control Region (GCR) reinforces the effect of another enhancer, Prox, that drives the expression of the genes *Lnp *and *Evx-2 *- both adjacent to the 5' end of the *HoxD *cluster - and the posterior *Hoxd *genes (*Hoxd-13 *to *Hoxd-10*) in the distal limb and genital buds (digit enhancer); it also regulates the expression of *Lnp *and *Evx-2 *in the central nervous system (neural enhancer) [19-21]. The action of the GCR in the nervous system is somehow restricted by boundary elements between *Evx-2 *and *Hoxd-13*, while in the limb bud its effect decreases progressively with distance from the 5' end of the *HoxD *cluster [22-24].

Defects in *Hoxd *genes or gene regulation often have an effect on limb development. For example, the mouse *Ulnaless *mutation causes reduction of the zeugopod, which is the result of the alteration of *Hoxd *gene expression due to an inversion of the *HoxD *cluster and subsequent change of *cis*-regulatory control [19,25,26]. Similar phenotypes can be observed in human mesomelic dysplasia patients, which results from microduplications in the *HoxD *cluster [27]. Normal limb development can also be affected by mutations within *Hoxd *genes. For example, the expansion of a poly-alanine tract in HOXD13 results in the synpolydactyly syndrome, with abnormal reductions, duplications and fusions of digits [28].

Despite their differences in limb types, frogs and salamanders are characterized by the presence of only four fingers. Of all amphibians, caecilians (Gymnophiona) probably form the most enigmatic order, as most of them spend their life hidden under the ground. Similar to snakes, they have an elongated trunk and have undergone secondary loss of limbs. The diversity in body plan and limbs among the three amphibian orders may have been affected by changes in *Hox *gene sequence, or, more likely, regulation (e.g. [29]).

At present, the only amphibian genome sequence publicly available is from the frog *Silurana tropicalis*. We constructed a BAC library of the aquatic caecilian *Typhlonectes natans *to obtain the sequence of the posterior end of the *HoxD *cluster, including *Evx-2*, *i.e*. the part of the cluster that is important in limb development and which has been shown to be under control of the Global Control Region. Comparative analyses of this region with the orthologous region of other vertebrates and the subsequent identification of conserved, non-coding, putative regulatory elements may shed light on the evolution of the caecilian body plan.

## Results and Discussion

### BAC sequencing and annotation

The haploid genome size of *Typhlonectes natans *was estimated at 13.37 pg by flow cytometry with chicken erythrocyte nuclei as an internal standard (data not shown). A pooled BAC library of about 460,000 clones with an average insert size of 107 kb from *Typhlonectes natans *was constructed, comprising a theoretical 3.8 × coverage of the genome, and screened by PCR to isolate a clone that contained the posterior *HoxD *cluster. A single clone with an estimated size of approximately 115 kb containing *Hoxd-13 *was sequenced using 454 sequencing technology [30] and over 15,000 reads were assembled into two supercontigs. The orientation of the two supercontigs was determined by sequencing of both BAC ends, and they were assembled into one final contig with a small gap arbitrarily set at 100 base pairs (bp) because the total sequence length is consistent with the estimated insert size. The total caecilian sequence comprises 116,633 bp, including a 100 bp gap (GenBank HQ398255). An initial blastx analysis and the software GenomeScan [31] identified the genes *Evx-2*, *Hoxd-13*, *Hoxd-11 *and a large part of *Hoxd-10*. The exon-intron boundaries were refined manually by alignment with *Hox *sequences of other vertebrates. *Hoxd-12 *was not found in these analyses.

### Vertebrate posterior *HoxD *cluster comparison

**Figure 1:**
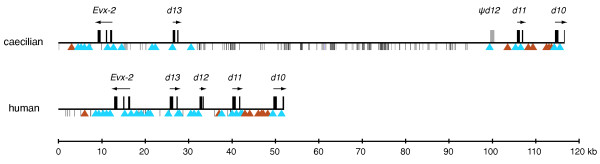
**Posterior *HoxD *cluster architecture of the caecilian**. The homologous region of the human is drawn for comparison. The exons of the genes are characterized by black boxes above the cluster (pseudogene in grey); arrows indicate the direction of transcription. The triangles below the cluster represent conserved non-coding regions (putative *cis*-regulatory elements in blue, potential ncRNA genes in orange) identified by the tracker program, bars and rectangles below the clusters denote repeats. Interspersed repeats identified by Censor are colored dark grey, other repeats, *i.e. *inverted and direct repeats that are not a part of known transposable elements, are light grey. All distances are drawn to scale.

We compared the posterior *HoxD *cluster of the caecilian with the clusters of the horn shark, zebrafish, coelacanth, frog, chicken, anole lizard, opossum, dog, mouse and human. In the caecilian, the 5' end of the *HoxD *cluster, starting from the stop codon of *Evx-2*, situated on the reverse strand, until the stop codon of *Hoxd-10 *is 107 kilo base pairs (kb), which is over 2.5 times larger than the orthologous region in mammals (approximately 40 kb) (Figure 1). Apart from the caecilian, the coelacanth and the lizard sequences are also larger than average, with lengths of 82 kb and 95 kb, respectively. The expansion of the caecilian *HoxD *cluster is mainly due to the lengthening of the intergenic region between *Hoxd-13 *and *Hoxd-11*, which is over six times larger than in the human. In the coelacanth, the intergenic region between *Evx-2 *and *Hoxd-12*, where the *Hoxd-13 *gene was lost, is almost four times longer than the corresponding region in mammals. In the anole lizard, not only the *HoxD *cluster, but also the other three *Hox *clusters are significantly longer [15].

**Figure 2:**
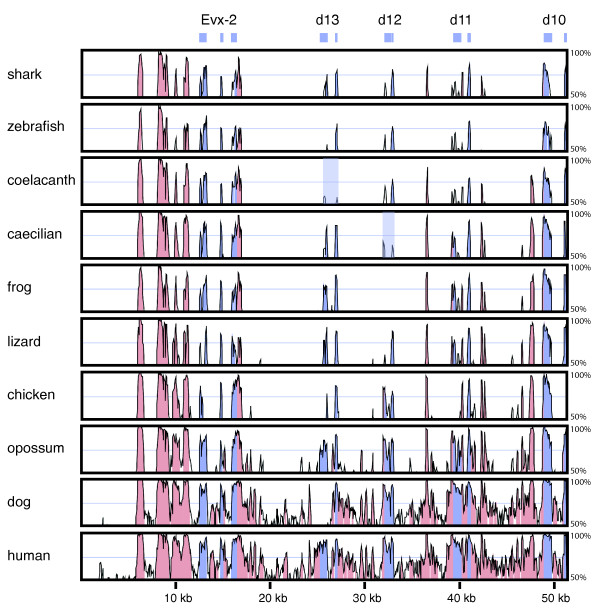
**Global alignment of the vertebrate posterior *HoxD *clusters**. The graphs represent the percentage of nucleotide sequence conservation of each vertebrate *HoxD *cluster compared to the orthologous region in the mouse *HoxD *cluster. Blue peaks represent coding regions, corresponding to the *Hox *exons on top of the figure; pink peaks represent highly conserved non-coding sequences. The shaded boxes indicate a *Hoxd-13 *pseudogene in the coelacanth and a *Hoxd-12 *pseudogene in the caecilian.

Global alignment with the other vertebrates uncovered the remains of *Hoxd-12 *in the caecilian (Figure 2). The presence of multiple frameshift-producing indels and stop codons implies that it is no longer protein coding and has become a pseudogene (*ψHox-d12*). As frogs and caecilians comprise the basal split within amphibians [32], the most parsimonious explanation for the absence of *Hoxd-12 *from the *Silurana tropicalis *genome and its pseudogenization in the caecilian is an early loss in amphibian evolution with unequal rates of evolution in *T. natans *and *S. tropicalis*, although two independent loss events cannot be excluded. We also discovered a pseudogene (*ψHoxd-13*) in the coelacanth posterior *HoxD *sequence. A blastx analysis revealed fragments of both exons that could still be aligned with other vertebrate *Hoxd-13 *genes.

The length of the coding sequences of *Hox *genes is similar in both amphibian species, except for *Hoxd-13*, which is over 100 bp shorter in *Typhlonectes*. In general, most posterior *HoxD *coding sequences have comparable lengths in all species used in this study, except for *Hoxd-13 *and *Hoxd-11*, which are about 25% longer in amniotes and placental mammals, respectively. The amniote or mammalian expansion of the HOXD13 and HOXD11 proteins can be attributed to sequences rich in alanine, glycine, serine and proline. In HOXD13, a homopolymeric repeat consisting of nine alanines in the chicken and 15 alanines in mammals [28] is not present in any other species. HOXD13 proteins are overall rich in serine and alanine in mammals, but not in non-amniotes. In addition to the conservation of both exons of each *Hox *gene, evolutionarily conserved regions were also detected in non-coding intergenic and intronic sequences. These conserved, non-coding sequences or phylogenetic footprints may represent regulatory elements and non-coding RNA genes and were further investigated.

### Distribution of repeats

To investigate whether the caecilian *HoxD *cluster expansion was accompanied by an accumulation of repeats, we compared it to the Repbase database [33] of known vertebrate repetitive elements using RepeatMasker [34] and Censor [35]. RepeatMasker identified 4.74% of the sequence as part of transposable elements, while Censor reports 7.45% interspersed repeats, mostly retrotransposons like LINES and SINES. It must be noted that the hits are not always very strong because of short alignment length. However, blastn searches of all the repeats resulted in a significant match with known repeats (e-value ≤ 1e-05) for 11 out of 54 repeats. Moreover, blastx searches of the intergenic regions recovered one additional SINE and one LINE. The majority of the repeats is located in the intergenic region between *Hoxd-13 *and *ψHoxd-12 *(Figure 1). The same analyses were performed for the other vertebrate sequences by comparison with the repeat databases for the respective species, if available. In the caecilian and the coelacanth, the repeats are almost exclusively found in the large intergenic region where the pseudogene is situated, *i.e. *between *Hoxd-13 *and *ψHoxd-12 *in the caecilian and between *Evx-2 *and *ψHoxd-13 *in the coelacanth. In the lizard and zebrafish, transposable elements are dispersed over the cluster, with a concentration of repeats between *Evx-2 *and *Hoxd-13 *in the latter. Additionally, a self-self blastn analysis was performed to identify direct and inverted repeats that are not part of known transposons. This revealed the presence of 30 inverted repeats (stem-loop), 15 palindromes (stem) and 12 direct repeats (minimum identity of 70%, e-value ≤1e-5) in the caecilian *HoxD *cluster. Again, almost all repeats are located in the region between *Hoxd-13 *and the *ψHoxd-12*. Some of these repeats are a part of transposable elements, but the majority is unknown. A similar result was obtained for the coelacanth sequence. Here, no direct repeats, 21 inverted repeats and 11 palindromes were found, all located in the region between *Evx-2 *and *Hoxd-12*, with several large stretches in the former intron of *ψHoxd-13*. The anole lizard sequence also contains a large amount of inverted and direct repetitive sequences, but spread across the cluster, which is in concordance with the distribution of transposable elements. Few or no additional repeats were found in the other vertebrate *HoxD *sequences. The presence of interspersed, repetitive DNA may undermine genomic stability [36]. In vertebrate genomes, regions containing developmental genes, such as *Hox *clusters, are usually devoid of transposable elements [37], suggesting the presence of a constraint against the invasion of foreign elements into a region essential for development. The caecilian, coelacanth, anole lizard and zebrafish have a higher than average amount of repetitive elements in the posterior *HoxD *cluster, and the caecilian and coelacanth also experienced the loss of a functional *Hox *gene. Whether the presence of repeats attributed to the pseudogenization of *Hoxd-12 *in the caecilian (and of *Hoxd-13 *in the coelacanth), or whether the loss of *Hoxd-12 *weakened the constraint and allowed repeats to accumulate, remains unknown. In the human genome, pseudogenes are frequently found in the vicinity of long inverted repeats [38].

Despite being essential for proper embryonic development, the loss of one *Hox *gene does not necessarily have a negative impact on an organism's phenotype, as *Hox *genes can be functionally equivalent [39]. However, expansion of the cluster may have an effect on gene regulation. Since all the genes in this region of the *HoxD *cluster are under the control of the long-range enhancer GCR, the loss of *Hoxd-12 *accompanied with the enlargement of the distance between *Hoxd-13 *and *Hoxd-11 *and the accumulation of repetitive elements in the caecilian may have an influence on the effect of the GCR on the expression of *Hoxd-11 *and *Hoxd-10*. Whether this effect, if any, is reflected in morphology, is unknown, but it is worth mentioning that a similar cluster architecture was found in the corn snake [16]. Some transposable elements have been exapted to modulate gene regulatory networks (reviewed by [40]). As changes in - especially developmental - gene regulation may lead to morphological changes [41], the adoption of a highly derived body plan, such as in caecilians, may have been facilitated by transposable elements. It is possible that caecilians and snakes have employed a similar mechanism of limb loss, though this is probably not the case in other limbless squamates, since *Hoxd-12 *is present in the slowworm *Anguis fragilis *[16].

### Identification of phylogenetic footprints

More and more regulatory sequences and non-coding RNA (ncRNA) genes are being discovered in the portion of the genome that does not code for proteins [42]. Although it has been shown that not every regulatory sequence is evolutionarily conserved and that an apparent function cannot always easily be allocated to a conserved sequence [43,44], screening genomes for evolutionarily conserved non-coding sequences is a widely used strategy to discover potential regulatory elements. Such elements are expected to be present in the vicinity of transcription factors or developmental genes, such as *Hox *genes [42]. We identified 33 evolutionarily conserved non-protein coding sequences, or so-called phylogenetic footprints, with the software tracker [45] (Additional file 1). To distinguish putatively transcribed footprints, we blast searched all footprints against the NCBI database of ESTs, which resulted in the identification of eight footprints between 29 and 876 bp long, not located within untranslated regions (UTRs), with one or more EST matches (Additional file 2). Because EST data are absent for many organisms, including coelacanth and caecilians, we did not find ESTs corresponding to these footprints for every organism. However, given the high degree of sequence conservation and the finding of ESTs of each footprint in at least two different organisms, we believe it is possible that transcription and perhaps the function of these elements are conserved. Therefore, we consider these footprints to be putative ncRNA genes, and the other, non-transcribed footprints as potential *cis*-regulatory elements (Figure 1).

Non-coding RNAs are functional molecules that are not translated into a protein. Instead, they are involved in post-transcriptional modification or DNA replication or have a regulatory function, and can be found in intergenic regions, introns and even in the UTR of genes or overlapping with protein coding genes [46]. The gene regulatory RNAs are usually small and act in *trans *by post-transcriptional silencing of target genes through the binding of complementary sites. Next to the large number of small ncRNAs, an increasing number of long ncRNAs is being described, which are at least 200 to over 10,000 nucleotides long (reviewed by [47]). Long ncRNAs can act independently of a target sequence, in *cis*, by interfering with the transcription of a neighboring gene, or in *trans*, by recruiting proteins that alter the chromatin state [47]. One example of a regulatory ncRNA in the *HoxC *cluster is HOTAIR, which epigenetically represses transcription of 40 kb across the *HoxD *locus [48]. Screening of all the expressed footprints against the Functional RNA Database [49] did not result in the identification of any classified ncRNA. However, four of them produced significant matches with putative RNAs predicted by Evofold, which is a method to identify functional RNA structures in vertebrates by using a combined probabilistic model of RNA structure and sequence evolution [50]. In addition, in footprint fp1, also known as CR3 [51], a significant RNA secondary structure was predicted (p = 0.92). Seven of the expressed footprints lie in the intergenic region between *Hoxd-11 *and *Hoxd-10 *(Figure 1). What tracker considers to be multiple footprints may correspond to a single potential ncRNA gene, as indicated by alignment of the region between *Hoxd-11 *and *Hoxd-10 *with the corresponding ESTs (not shown). Two of the expressed footprints were previously identified as regulatory regions RVIII/RIX (fp26) and RX (fp20) ([52-55]. The fact that these two regulatory regions appear to be transcribed may shed new light on how they function.

*Cis*-regulatory elements can be anticipated in intergenic regions, in introns and in the 5' and 3' UTR of protein-coding genes, and may act as a promoter, enhancer, repressor or insulator [42]. In total, 24 footprints (fps) are putative *cis*-regulatory elements. Nine of these were found in the intergenic regions, including the area downstream of *Evx-2*; four footprints are located in an intron, five located in the 5' UTR and six located in the 3' UTR of the *Hox *and *Evx-2 *genes. Three of the intergenic footprints were identified in previous studies and are known as RXI (fp18) and RXII (fp9 and fp10) [23,56]. Two other intergenic footprints (fp 2 and fp3) are extremely conserved in all species used in this study and are located downstream *Evx-2*. The reason for this high degree of conservation however remains unclear [51]. One footprint (fp23) that was found in the intron of *Hoxd-11 *contains a HB1 element, which consists of homeodomain binding sites. This element is previously described from the intron of *Hoxa7*, the *Drosophila *homolog *Ubx*, the introns of *Hox4 *genes and the intron of *Hoxa-11 *[57-60]. Of the five footprints in the 5'UTR, two also include the promoter region. The 5' and 3' UTRs of mRNAs play an important role in the post-transcriptional regulation of gene expression through the presence of *cis*-acting elements and through interaction with micro-RNAs (miRNAs) [61,62]. Conserved sequences in 3' UTRs may contain potential target sites for miRNAs that are involved in post-transcriptional gene silencing. Micro-RNAs are short, single-stranded RNA molecules of ~22 nucleotides that show at least partial complementarities to their target mRNA. If the miRNA is only partially complementary, a perfect match between the seed (nucleotides 2 to 7) of the miRNA and the target mRNA is necessary for inhibition of translation or for promotion of deadenylation [63,64]. Therefore all six footprints located in the 3'UTR of the genes were screened for the presence of hypothetical target sites for miRNAs. One footprint (fp15), located in the 3'UTR of *Hoxd-13*, contains a short motif of seven nucleotides, with perfect match to the seed of the miRNA miR-26.

### Reconstruction of conserved element evolution

**Figure 3:**
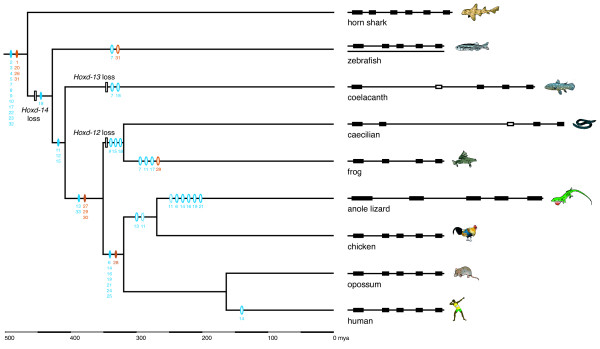
**Reconstruction of vertebrate posterior *HoxD *cluster evolution**. A Dollo parsimony reconstruction of the evolution of *HoxD *cluster elements shows the ancestral presence of 16 conserved elements in jawed vertebrates. Divergence times of the species are taken from Alfaro et al. [73]. A schematic representation of the posterior *HoxD *cluster with correct relative distances is shown on the right. The human cluster is representative for placental mammals, except for the loss of footprint 14, which did not occur in other mammals. Potential ncRNAs are symbolized by orange ovals, putative *cis*-regulatory elements by blue ovals. Gain of elements is shown as full ovals, with the respective footprint number underneath, open ovals and bars represent the loss of a footprint and *Hoxd *gene, respectively. Ovals with dotted lines denote equivocal reconstruction of footprint 11, which is due to missing data in the chicken sequence. Gains and losses of footprints were not dated.

Gains and losses of putative ncRNAs and *cis*-regulatory elements in vertebrate posterior *HoxD *clusters were mapped on a vertebrate timescale under the Dollo parsimony criterion, *i.e. *assuming a single origin (Figure 3). Two putative *cis*-regulatory elements have been lost in the amphibian ancestor: fp9, which is one of two motifs of RXII, and fp15, a small footprint in the 3' UTR of *Hoxd-13*. The conserved region RXII is a boundary element located in the intergenic region between *Evx-2 *and *Hoxd-13*, whose promoters are in each other's vicinity. Unlike *Hoxd *genes, *Evx-2 *is expressed in the central nervous system, regulated by the neural enhancer of the GCR. RXII is considered to function as an insulator to prevent the ectopic expression of *Hoxd *genes in the nervous system, since GCR regulation is not promoter-specific [23]. Although tracker failed to recover the entire RXII element (fp9 and fp10) in the zebrafish, short, apparently homologous sequences were found in the zebrafish cluster in the same area as in other vertebrates [23]. Moreover, both footprints were found in the horn shark, which points to their ancestral presence in vertebrates, and therefore we conclude that fp9 was lost in amphibians. Footprint 15 is a short DNA stretch of ~22 bp with a highly conserved motif of 7 bp located in the 3' UTR of *Hoxd-13*. This motif is somewhat degenerated in the coelacanth, which does not have a functional *Hoxd-13 *gene, and it is located around 14 kb 5' to *ψHoxd-13*, which may indicate that this sequence is not homologous to the other vertebrate fp15 sequences. On the other hand, pseudogenization of *Hoxd-13 *and insertion of repetitive sequences may have promoted the relative relocation of this element. For our reconstruction, we considered all footprints as identified by the tracker software to be truly conserved sequences. Therefore, we conclude that this element originated in Sarcopterygii, and was lost in amphibians. In theory, this footprint can serve as a target for the microRNA miR-26, which is expressed in neurons and astrocytes of the developing mouse brain [65]. The absence of both elements may indicate that restriction of *Hoxd-13 *expression in neural tissue is regulated differently in amphibians.

## Conclusion

The posterior *HoxD *cluster of the caecilian is much larger than that of most known vertebrates due to the accumulation of interspersed and inverted repeats accompanied with *Hoxd-12 *gene loss. A similar event occurred in the coelacanth and in the corn snake *HoxD *cluster. Whether these occurrences are reflected in the morphology of these species is not clear, but it is possible that caecilians and snakes adopted a similar mechanism that resulted in body elongation and limb loss. The distance created between two subsequent genes that are under the control of the same long-range limb enhancer is likely to have some effect on the action of this enhancer on the genes after the void. A number of conserved, non-coding regions have been identified in the *HoxD *cluster, some of them showing extremely high conservation among all vertebrates. While no new footprints arose in the amphibian lineage, at least one, and possibly two conserved sequences were lost. These results, together with data on other vertebrate *Hox *clusters show that tetrapod *Hox *clusters show more variation than expected previously.

## Methods

### Genomic library and DNA sequencing

High molecular weight DNA was extracted from erythrocytes from two *Typhlonectes natans *specimens and was used to construct a BAC library according to Osoegawa et al. [66] and as described by Danke et al. [67]. The genome size was estimated by flow cytometry analysis using chicken erythrocyte nuclei as a standard. EcoRI partial digests of the caecilian DNA were size selected and inserted in the pCC1 BAC vector (Epicentre) and the library was combined in 2304 pools containing 200 clones each. The library was screened by PCR with primers specific for *Hoxd-13 *(forward primer: 5'-GCAATGAAGGCGCCTCCAG-3', reverse primer: 5'-GGAGATATAGGTGTCGTGCCTCGG-3') to isolate the posterior end of the *HoxD *cluster. The positive clone was 454 GS FLX-sequenced and assembled by Eurofins MWG Operon (Ebersberg, Germany). The clone was also end sequenced using BigDye 3.1 chemistry on an ABI 3100 Genetic Analyzer. Several smaller contigs were assembled manually and contig overlaps were confirmed by PCR, except in one case where a gap was present. Because the length of the two contigs is consistent with the size of the insert, we inferred this gap to be small and therefore arbitrarily set it at 100 bp.

### Caecilian *HoxD *cluster annotation

The genes in the caecilian posterior *HoxD *cluster were annotated by initial blastx searches of the entire cluster sequence and with the program GenomeScan [31] using mouse HOXD and EVX2 proteins as a training set. Exon - intron boundaries were determined manually by alignment with *Hox *sequences of other vertebrates.

### *HoxD *alignments

Global alignments of the caecilian posterior *HoxD *cluster with other vertebrate *HoxD *clusters were performed with MultiPipMaker [68] and VISTA [69]. The *HoxD *clusters of the following species were used: *Heterodontus francisci *(horn shark, AF224263), *Danio rerio *(zebrafish, UCSC Genome Browser) *Latimeria menadoensis *(Indonesian coelacanth, FJ497008), *Silurana tropicalis *(tropical clawed frog, JGI), *Gallus gallus *(chicken, ENSEMBL), *Anolis carolinensis *(green anole, UCSC Genome Browser), *Monodelphis domestica *(grey short-tailed opossum, ENSEMBL), *Canis familiaris *(domestic dog, ENSEMBL), *Homo sapiens *(human, NT_005403) and *Mus musculus *(mouse, AC_015584). The MultiPipMaker alignments were performed with mouse and *T. natans *as reference sequence respectively.

Interspersed repeats and low complexity regions in all clusters were masked by screening against a library of repetitive elements if available for the organism by RepeatMasker [34]. If no such library was available, as for the horn shark, coelacanth, caecilian, lizard and opossum, the sequences were compared to a database of transposable element encoded proteins.

### Repeat content

The repeat content of the *HoxD *clusters was determined with Censor [35] and RepeatMasker, using the Repbase library of the species-specific or vertebrate repeats and a database of transposable element encoded proteins. All reported interspersed repeats were taken into account. In addition, self-self blastn analyses were performed to identify direct and inverted repeats within each cluster. Only repeats with maximum 30% mismatch and an e-value ≤ 1e-05 were retained.

### Analyses of phylogenetic footprints

The program tracker [45] was used to detect evolutionarily conserved non-coding sequences or phylogenetic footprints. This program is based on blastz [70] to produce initial local pairwise alignments of all pairs of the input sequences. Only the intergenic regions between two homologous genes are compared. After several filtering steps, these alignments, which contain a window of 12 nucleotides with minimum identity of 75%, are assembled into groups of partially overlapping regions, resulting in local sequence alignments, or footprint cliques. To be able to detect conserved sequences in all non-coding regions, introns were treated as intergenic regions. This analysis was repeated using the same, but repeat masked sequences and all footprints from both analyses were combined. Not every footprint is necessarily conserved in each taxon, as its presence or absence may be indicative of the loss of a *cis*-regulatory element and a subsequent change in gene regulation. Phylogenetic footprints were treated as two classes: potential (*cis*-) regulatory elements and putative non-coding RNA genes. Conserved sequences found in the 3' UTR of genes were screened for the presence of hypothetical target sites of miRNAs using TargetScan Release 5.1 [63,71]. To detect whether footprints are expressed and thus may be ncRNA genes, a blastn search of all footprints against the NCBI database of ESTs was performed. Footprints with one or more EST matches were screened against the Functional RNA Database [49] for similarity with known ncRNAs. In addition, the RNAz server (Vienna RNA server, http://rna.tbi.univie.ac.at) was used to identify thermodynamically stable and evolutionarily conserved RNA secondary structures in the footprint alignments.

A Dollo parsimony reconstruction of the genes and footprints was done with MacClade v4.06 [72] to assess whether putative *cis*-regulatory elements and ncRNAs were lost or gained during vertebrate evolution.

## Authors' contributions

The research was designed by AM and FB. AM conducted the laboratory work, except the 454 sequencing; CTA provided assistance with the BAC library construction. AM performed the analyses. All authors wrote the manuscript.

## Supplementary Material

Additional file 1 **Summary of the conserved sequences in vertebrate *Hox *clusters**. This table gives an overview of the positions of the coding regions in the vertebrate sequences as well as all footprint positions identified by the tracker software. The footprints are named fp1 to fp33, and footprints that are expressed in at least two species are indicated in bold. Click here for file

Additional file 2 **EST blast hits of expressed footprints**. All ESTs that correspond to the expressed footprints are given with Genbank accession number, species name and tissue source. Click here for file
